# Moderation of the association between COVID-19-related income loss and depression by receipt of financial support: Repeated cross-sectional surveys of young adults in Canada and France (2020–2021)

**DOI:** 10.1016/j.ssmph.2023.101340

**Published:** 2023-01-11

**Authors:** Pierre-julien Coulaud, Travis Salway, Julie Jesson, Naseeb Bolduc, Olivier Ferlatte, Karine Bertrand, Annabel Desgrées du Loû, Emily Jenkins, Marie Jauffret-Roustide, Rod Knight

**Affiliations:** a British Columbia Centre on Substance Use, Vancouver, British Columbia, Canada; b Department of Medicine, University of British Columbia, Vancouver, British Columbia, Canada; c Faculty of Health Sciences, Simon Fraser University, Burnaby, British Columbia, Canada; d British Columbia Centre for Disease Control, Vancouver, British Columbia, Canada; e Centre for Gender and Sexual Health Equity, Vancouver, British Columbia, Canada; f School of Population and Public Health, University of British Columbia, Vancouver, British Columbia, Canada; g École de Santé Publique de l’Université de Montréal, Montreal, Quebec, Canada; h Centre de Recherche en Santé Publique, Université de Montréal et CIUSSS du Centre-Sud-de-l’Île-de-Montréal, Montreal, Quebec, Canada; i Faculty of Medicine and Health Sciences, Université de Sherbrooke, Longueuil, Québec, Canada; j Centre Population et Développement, Université Paris Cité, Institut de Recherche pour le Développement, Inserm, Paris, France; k School of Nursing, University of British Columbia, Vancouver, British Columbia, Canada; l Centre d'Étude des Mouvements Sociaux (EHESS/CNRS UMR8044/INSERM U1276), Paris, France; m Baldy Center on Law and Social Policy, Buffalo University, NY, USA

**Keywords:** Young adults, COVID-19, Financial support, Depression

## Abstract

**Background:**

To mitigate the adverse effects of the COVID-19 pandemic on financial resources, governments and family/friends mobilized financial support interventions (e.g., emergency aid funds) and assistance. However, little is known about how financial assistance alleviated mental health problems. This study aimed to investigate the moderating effect of financial support from the government or from family/friends on the association between income loss and depression among young adults.

**Methods:**

Two online cross-sectional surveys among young adults ages 18–29 living in Canada and France were conducted in 2020 (n = 4,511) and 2021 (n = 3,329). Moderate-to-severe depressive symptoms were measured using the Patient Health Questionnaire-9 (cut-off score**:** ≥10). Two logistic regression models were performed for each survey with an interaction term between income loss and financial support (government or family/friends modeled separately), controlling for demographics.

**Results:**

Overall, half reported depressive symptoms (2020/2021: 53.5%/45.6%), and over a third lost income (2020/2021: 10.2%/11.6% all income, 37.7%/21.6% some income). In 2020, 40.6% received government financial support (17.7% in 2021) while family/friends support was received by 12% (in both surveys). In both surveys, among those who received governmental financial support, income loss was associated with depression, whether participants lost all their income (e.g., 2020: Adjusted Odds Ratios (AOR) 1.75, 95% Confidence Interval [1.29–2.44]), or some of their income (e.g., 2020: AOR 1.45 [1.17–1.81]). However, among those who received family/friends financial support, income loss was no longer significantly associated with depression in both cycles, whether participants lost all their income (e.g., 2020: AOR 1.37 [0.78–2.40]), or some of their income (e.g., 2020: AOR 1.31 [0.86–1.99]).

**Conclusions:**

Association between income loss and depression was moderated by receipt of family/friends financial support but not by receipt of government financial support. Financial support interventions may help to mitigate the negative effects of income loss on young adults mental health during periods of economic crisis.

## Highlights

• Young adults’ finances have been adversely affected by the COVID-19 economic crisis.
• Those who lost income and received family financial support had lower risk of depression.
• No protective effect was found for those who received governmental financial support.
• Adequate financial support may have a positive effect on youth mental health.

## 1 Introduction

The implementation of successive preventive public health measures (e.g., periods of lockdown, closures of non-essential businesses) over the first two years of the COVID-19 pandemic (2020 through 2021) had a critical impact on the financial resources of populations, globally. This has particularly been the case for young adults (i.e., 18–29 years), a group who are more likely to be employed in part-time and temporary jobs, and work in sectors hardest hit by COVID-19 restrictions, such as hospitality and food service sectors (Cook et al., 2021; International Labour Organization, 2021). Data from OECD survey reported that 24% of youth 15–24 years of age were working in heavily affected sectors in 2020 compared to 16% in older age groups (OECD, 2021a). A recent report from high-income countries showed that the drop in employment levels between 2019 and 2020 was five times greater among youth (15–24), relative to older age groups (O’Higgins & Verick, 2021). The context of the COVID-19 pandemic also reduced opportunities related to job search and availability of work for young adults during a critical life stage of entry into the labour market (OECD, 2021c). Qualitative explorations have indicated that these economic disruptions represented major concerns for young adults who reported increasing feelings of precarity, financial strain, and uncertainty about their career development (Burgess et al., 2022; Lindsay & Ahmed, 2021). These experiences of emotional stress due to the COVID-19-induced economic crisis represent critical risk factors for mental health challenges (Achdut & Refaeli, 2020; de Miquel et al., 2022; Graupensperger et al., 2022; Ranta et al., 2020; Shanahan et al., 2022), including depression, the prevalence of which has steadily increased among young adults in recent decades (Mojtabai et al., 2016). Previous studies have also showed that young adults with lower family income (Basheti et al., 2021; Browning et al., 2021; Yang & Yang, 2022), and those who experienced income loss, job insecurity or unemployment due to the pandemic were more likely to report depressive symptoms (Ganson et al., 2021; Melchior et al., 2022; Thorndike et al., 2022). These findings underscore the need to further investigate the role of financial support strategies on mental health outcomes among young adults.

To mitigate the financial difficulties experienced during the COVID-19 pandemic, including among young adults, a number of governments around the world launched or bolstered financial support interventions ranging from financial assistance, emergency aid funds for students, extension of employment insurance programs, and increased pension payments for those with pre-existing health conditions (e.g., disability) (OECD, 2021b). Furthermore, family and friends also provided financial assistance, including for young adults who faced disproportionate financial burdens related to the pandemic (Lindsay et al., 2021; McMaughan et al., 2021; McPherson, 2020). While both governmental and personal (i.e., family, friends) financial support interventions may have a positive impact on financial resources, less is known about the additional benefits of these interventions in alleviating mental health concerns among young adults, particularly during periods of economic instability induced by pandemic-related containment measures.

Previous evidence suggests that the implementation of public compensation for pandemic-induced losses may have led to a decline in negative mental health outcomes. For example, an ecological study conducted in Latin America investigating mental health-related Google searches during COVID-19 lockdowns showed a substantial decrease for insomnia and suicide searches after the passage of income support programs, while no such beneficial association was observed for depression-related searches (Silverio-Murillo et al., 2021). A global survey from 19 countries (mainly in high-income countries) found a lower number of helpline calls due to fear, loneliness, and suicidality in countries that implemented more generous income support interventions (Brülhart et al., 2021). Another survey conducted between April and July 2020 among students in high- and upper-middle-income countries provided a more nuanced interpretation with no significant effect of economic support interventions (i.e., income protection, debt or contract relief) on depressive symptoms (Buffel et al., 2022). The authors explained this result by the fact that such interventions may have a positive effect on mental health in the long-term (i.e., requiring longer follow-up periods to detect an effect) and may have more impact among those working in particular sectors that have been critically affected by the COVID-19 containment measures (i.e., requiring industry-specific sub-group analyses) (Buffel et al., 2022). All of these studies investigated the role of financial support interventions using an overall measure of financial assistance to households (i.e., economic index from the Oxford COVID-19 Government Response Tracker) (Hale et al., 2021), which does not necessarily indicate whether or not young adults were able to benefit from these interventions at an individual level.

**Fig. 1:**
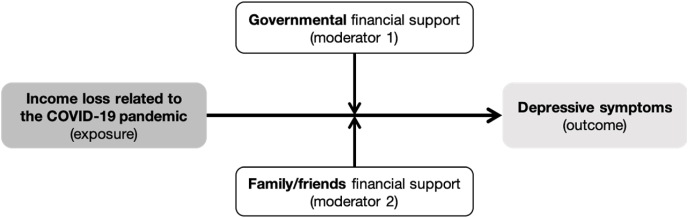
Conceptual diagram of hypothesized relations between income loss due to the pandemic, governmental or family/friends financial support, and depressive symptoms.

In the present article, we therefore use self-reported financial measures from a large and diverse sample of young adults living in Canada and France to assess whether financial support interventions from the government or from family and friends may mitigate the effect of income loss on depressive symptoms. As illustrated in Fig. 1, we hypothesized that financial support interventions would moderate the relationship between exposure to income loss due to COVID-19 pandemic-related restrictions and depressive symptoms, leading to a reduction in the odds ratio among those who received supports. In addition, we hypothesized that personal support would have a greater moderating effect on this relationship (compared to the financial support from governmental funds) because this financial support may be embedded in a trusted and reliable environment that provides appropriate and timely support in line with the financial needs of young adults, as well as a therapeutic benefit of social support or support in finding mental health services.

## 2 Material and methods

### 2.1 Study design and settings

This present analysis is a sub-analysis of data collected from the *France Canada Observatory on COVID-19, Youth health and Social well-being (FOCUS)* study (www.focus-study.me), an international mixed-methods research study investigating the effect of the COVID-19 pandemic on the social and health outcomes of young adults living in Canada and France through a series of repeated annual cross-sectional online surveys and qualitative interviews. Specifically, for this analysis, we used data from the two first online surveys conducted among young adults living in Canada and France. The first online survey was conducted during the second wave of the COVID-19 pandemic from 8 October to 23 December 2020, referred to herein as the “Fall 2020 survey”. The second online survey was launched during the fourth wave between July 4 and 13 December 2021, referred hereafter as the “Summer/Fall 2021 survey”.

#### 2.1.1 COVID-19 history and public health responses

Overall, Canada and France are two high-income countries that have been significantly affected by the COVID-19 pandemic, with more than 2 and 10 millions cases, and more than 30,000 and 123,000 deaths, respectively, between January 2020 and December 2021 (Ritchie et al., 2020). During this period, Canada and France experienced four successive COVID-19 waves (i.e., Spring and Fall of 2020, Winter 2021, and Summer of 2021). In both countries, young adults ages 20–29 represented about one fifth of the total COVID-19 cases over the period 2020–2021 (Government of Canada, 2021b; Santé Publique France, 2021).

In Canada, the COVID-19 pandemic started in late January 2020 and reached a first peak in March 2020, with most of the cases reported in the two most populous provinces of Québec and Ontario (Government of Canada, 2021b). In response and during the first wave, the federal, provincial, and territorial governments implemented a range of containment measures and recommendations including physical distancing measures, closures of businesses (e.g., ‘non-essential’ service industry), closures of schools and universities, working from home, and restrictions on travelling and social gatherings (Boire-Schwab et al., 2021). In May 2020, each province and territory implemented detailed reopening plans that consisted of a series of phases to gradually ease restrictions, including reopening businesses to allow indoor activities with masking, with physical distancing and capacity limits remaining in place. The increased number of COVID-19 cases in September 2020 prompted federal and provincial health authorities to re-implement preventive measures (e.g., avoiding non-essential travel, limiting social gatherings, lockdowns, and business restrictions) to mitigate the spread of the pandemic. For instance, provincial governments of Québec and Ontario re-introduced a series of business restrictions that particularly affected service industries, such as closure of indoor dining and non-essential construction work, as well as capacity limits for non-essential retailers (Institut national de santé publique du Québec, 2022; Stikeman Elliott, 2022). The second wave of the pandemic peaked in early January 2021 and the third in mid-April 2021, with more than 20 cases per 100,000 people per day (Government of Canada, 2021b). The restrictions limiting travel and gatherings were mostly lifted by early June, in response to a decline in COVID-19 infections and increased vaccination (i.e., 70% of the Canadian population were fully vaccinated at the end of July 2021 (Government of Canada, 2022)). The fourth wave was observed in late summer, mostly in September before Canada was hit by the fifth wave due to the Omicron variant in December 2021.

In France, a first nationwide lockdown was implemented from mid-March to mid-May 2020 in response to the first wave of the COVID-19 pandemic. The government of France then gradually eased the containment measures, starting with the reopening of primary schools, restaurants and shops, until travel restrictions were lifted as of the end of June 2020. The second wave started in August 2020 and reached a peak in early November with more than 100 cases per 100,000 people per day (Santé Publique France, 2022). In mid-October 2020, nighttime curfews were imposed in metropolitan areas, and a second nationwide lockdown was introduced at the end of October with closures to non-essential services and travel restrictions. At this time, schools and universities remained open. After gradually lifting the second lockdown, maintaining curfews and selected measures (e.g., closures of restaurants, restrictions in social gatherings), a third partial lockdown was implemented in March 2021, first regionally, and then nationwide. The third peak occurred in April 2021 with more than 70 cases per 100,000 people per day (Santé Publique France, 2022). Restrictions from the third lockdown were gradually lifted from May to June 2021. By mid-July 2021, France was facing a resurgence of cases due to the Delta variant (i.e., fourth wave) with more than 30 cases per 100,000 people per day in August (Santé Publique France, 2022). During the summer of 2021, the government of France introduced a “sanitary pass” (i.e., a proof of completed vaccination, recent infection or recent negative test) that was required to access restaurants, cultural venues, and health services, which adversely affected the capacity of the service industries (Gouvernement Français, 2021; Ward et al., 2022). In the meantime, vaccination rates increased, reaching 80% of the French population who had received complete vaccination in September 2021 (Ritchie et al., 2020).

#### 2.1.2 Financial support interventions

As in many countries across the globe, financial resources of young adults living in Canada and France were adversely impacted during the first two years of the COVID-19 pandemic. An OECD survey conducted in September–October 2020 indicated that 20% (Canada) and 10% (France) of young adults 18–29 years reported job loss in their household, while about half (60% in Canada and 40% in France) reported some form of job-related disruption, including job loss, reduced working hours, pay cut, and/or unpaid leave (OECD, 2021c).

In response to the COVID-19-related economic crisis, the government of Canada implemented a series of financial support interventions. Most of these interventions were temporary and designed to alleviate financial difficulties during the first year of the pandemic. For example, the Canada Emergency Student Benefit (CESB) was introduced between May and August 2020 and offered a Canadian Dollars (CAD) $1,250 a-month benefit (for a maximum period of 16 weeks) to post-secondary students and recent graduates who were unable to find work due to the pandemic (Government of Canada, 2020b). Applicants with dependants or disabilities were also eligible for an extra CAD$750 for each four-week period. Employed or self-employed individuals aged at least 15 years of age who were affected by the pandemic (e.g., job loss, reduced work hours) had the opportunity to apply to the Canada Emergency Response Benefit (CERB) for a CAD$2,000 a-month benefit between March and September 2020 (Government of Canada, 2020a). Other interventions were implemented to provide income support during lockdown periods (i.e., Canada Worker Lockdown Benefit (CWLB)), or for those who are unable to work because they were sick or had underlying health condition (i.e., Canada Recovery Sickness Benefit (CRSB)) or because they were providing care to their child or a family member (i.e., Canada Recovery Caregiving Benefit (CRCB)) (Government of Canada, 2021a). In addition, many provincial and territorial governments also provided financial supports to local youth communities, such as youth working in essential services (e.g., Incentive Program to Retain Essential Workers (IPREW) in Québec). Provincial authorities implemented temporary housing support programs (e.g., provincial rent subsidy) and specific funds were allocated to support businesses that have been critically affected by the pandemic (e.g., to avoid layoffs, to help keep staff on payroll) (Government of Canada, 2021a). The government of Canada also revised the minimum requirements and simplified procedures to increase accessibility to income supports, such as employment insurance (EI) benefits (Government of Canada, 2020c). EI sickness benefits provided up to 15 weeks of income replacement for a maximum of CAD$500 per week, and EI regular benefits were equivalent to 55% of the insurable earnings for up to a maximum of 45 weeks, depending on the province of residence. Furthermore, the accumulation of interest on Canada Student Loans was suspended until March 2023 (Government of Canada, 2022), and wage subsidies were provided to local enterprises to support summer jobs and internships for young people.

In France, several interventions were taken by the French government to extend pre-existing financial support for young people and increase social benefits for unemployed youth and those with a low-income background (Government of France, 2021). For example, a support of 200 Euros (€) was provided in June 2020 to nearly 800,000 French young people under the age of 25 who experienced financial difficulties due to the pandemic, as well as those who were not in education and those who received housing assistance. Students whose family experienced a significant loss of income due to the pandemic were able to apply for revision to the amount of their scholarship applications. In collaboration with university partners, the government of France provided a one-off emergency aid up to 500€ to university and post-secondary students who experienced financial hardship. Recipients of higher education scholarships in 2019–2020 and who were under the age of 30, searching for a job, and unemployed youth under the age of 26, were also eligible to apply for supports from employment services, equivalent to 70% of the amount of their scholarship. An additional benefit of 100€ was allocated to recipients who were not living in their family home. The French government also introduced housing assistance for young people to help them stay in their homes, including rent deferrals and freezes, and housing allowance (e.g., reduce rental cost, moving assistance programs). Since February 2021, young workers under the age of 25 who have been employed for less than 18 months (with a salary <1,400€ per month) can receive a one-off 1,000€ to support payments for housing (Action Logement, 2022). Partial unemployment insurance, corresponding to 70% of the previous gross earnings, was also provided to employed, and self-employed young adults who have been affected by the COVID-19 measures (e.g., those who lost their job, those suffering from COVID-19) (Ministère du Travail du Plein emploi et de l’Insertion, 2020; Sachs, 2020). To facilitate access to employment, the government of France made a significant investment in July 2020 (overall budget of 9 billion €) to launch a youth employment program called “1 jeune 1 solution” (www.1jeune1solution.gouv.fr) providing a package of interventions including hiring subsidies for youth under the age of 26 and for apprenticeship contracts, employment support (e.g., mentorship), and training opportunities for socially disadvantaged groups of youth (e.g., those who experienced poverty, disability).

### 2.2 Study recruitment and data collection

Using a non-probabilistic sampling approach, survey participants were recruited through online posts and advertisements on social media platforms (e.g., Facebook and Instagram) and on websites of university partners, through media articles, and word of mouth. Additional efforts were made to reach underrepresented populations and remote geographic areas by using targeted online ads from demographic criteria available on social media platforms (e.g., age, gender). Participants who participated in the Fall 2020 survey and provided their consent to be contacted for a follow-up survey were invited to complete the second survey in Summer/Fall 2021 in order to facilitate a nested online cohort. Given the limited number of participants who completed the two FOCUS surveys and reported income loss (n = 545), we were not able to use this longitudinal data to explore whether receiving financial support in Fall 2020 may influence the frequency of depressive symptoms reported in Summer/Fall 2021. Participants who completed both surveys were included in both analyses (i.e., 2020 and 2021).

To participate in the FOCUS surveys, young adults had to meet the following criteria: 1) be between the age of majority (18 or 19 in Canada, depending on the province or territory of residence; 18 in France) and 29 years; 2) reside in Canada or France; and 3) be able to complete the survey in English (Canada) or French (either country). All participants completed an online consent form prior to beginning the survey and were provided with an option to enter a draw to win one of three cash prizes (CAD$100 in Canada, 100€ in France). The questionnaire comprised four major sections: sociodemographic, social life and experiences during the pandemic (including questions about income loss and financial support), access to social and health services, and health outcomes (e.g., mental health). Survey data were collected using *Qualtrics*. A pilot study with 10 participants was conducted to assess the internal consistency of the questionnaires in both countries. Participants were able to withdraw from the survey at any time. Ethical approval was granted by the University of British Columbia Behavioural Research Ethics Board (H20-02053). Survey data was saved and stored in a secure server at the British Columbia Centre on Substance Use. In France, the Data Protection Officer at the University of Paris was consulted to ensure that our data privacy and security procedures were compliant with the European Union's General Data Protection Regulation (GDPR).

### 2.3 Study population

The population for the present study included participants of the FOCUS surveys who reported having received any income over the last year (i.e., from any source including employment insurance or any other official financial support) and who completed the questions about depression. Participants who did not have any income were excluded from this analysis.

### 2.4 Measures

#### 2.4.1 Outcome: depressive symptoms

Depressive symptoms were measured using the Patient Health Questionnaire-9 (PHQ-9) (Kroenke & Spitzer, 2002), a scale rating the occurrence and severity of nine depressive symptoms (derived from criteria of the Diagnostic and Statistical Manual of Mental Disorders, DSM-IV (Spitzer et al., 1999)) within the past two weeks. Each item is scored on a scale from “not at all” (=0) to “nearly every day” (=3). Total PHQ-9 scores range from 0 to 27. A cut-point of 10 was used to identify participants with moderate-to-severe depressive symptoms. This threshold was based on previously established thresholds suggesting that individuals scoring ≥10 should be referred to a mental health professional (Kroenke et al., 2001; Manea et al., 2012).

#### 2.4.2 Exposure: income loss related to the COVID-19 pandemic

Loss of income was assessed by asking participants the following question: “In the last 6 months, have you lost any income (including salary, employment insurance, government assistance …) because of measures taken as part of the COVID-19 pandemic?”. Participants were provided with three responses options in the Fall 2020 survey (“yes, some of my income”, “yes, all of my income”, “no”), and four response options in the Summer/Fall 2021 survey (“yes, some of my income (less than 50%)”, “yes, most of my income (more than 50%)”, “yes, all of my income”, “no”). Given that sociodemographic characteristics (described below) and prevalence of depressive symptoms among Summer/Fall 2021 participants who lost more than 50% of their income (n = 135, 4.1%) were not significantly different from those who lost all of their income (n = 249, 7.5%) (data not shown), these two groups were combined into one category to obtain similar number of categories between surveys.

#### 2.4.3 Moderator: financial support

Financial support was measured using a multiple-choice question that asked participants to whether or not they had received financial support because of the COVID-19 pandemic and to select the kind of financial support they received. Response options were as follows: “no”, “yes, from friends or family”, “yes, from youth organizations”, “yes, from COVID-specific emergency aid funds”. An additional open-text response was also provided to specify other sources of financial support. These open-text responses were re-categorized into one of the pre-determined options wherever possible. Given the limited number of participants who received financial support from youth organizations (n = 42, 0.9% in Fall 2020 and n = 49, 1.5% in Summer/Fall 2021), we did not include this support option in our analysis.

In the Fall 2020 survey, we also asked participants who had received financial support from COVID-19-specific emergency aid funds to specify the nature of these funds. In Canada, respondents were able to select CERB, CESB, provincial rent subsidy, funds for businesses, and other funds (open-text response). In France, the following propositions were included: exceptional solidarity fund (less than 25 years old and students), government assistance within the framework of a professional activity (employees, self-employed, shopkeepers, etc.), funds from universities, extra bonus for caregivers and mobilized employees, and other funds (open-text response).

#### 2.4.4 Sociodemographic covariates

Sociodemographic characteristics included in this analysis were: age, gender identity, trans identity (using the following questions “some people describe themselves as trans when their gender identity is different from the sex assigned to them at birth. Do you identify as trans?”), country and area of residence (large city of 100,000 or more people, medium city between 30,000–99,999 people, small city or rural of less than 29,999 people), educational attainment, employment status, living arrangements, and individual income in the past year (i.e., < or ≥ CAD$ 20,000, corresponding to a monthly income of 1,200€ in France). Data about ethno-racial identity was collected differently in the two countries. In Canada, ethno-racial identity was collected using the Canadian Institute for Health Information standards (Canadian Institute for Health Information, 2020). Participants who selected Indigenous (includes those who self-identify as First Nations, Métis, Inuk/Inuit descents) or those who reported any ethno-racial identity (one or more) other than white were classified as “racialized” persons. The category “non-racialized” includes young adults who selected “white” only. In France, where collecting data about ethno-racial identity is not permitted, we asked participants to report the country of birth of their parents and grand-parents from both sides (i.e., second and third generation of immigrants) to estimate the ethno-cultural origin of French participants as a proxy for ethno-racial identity (Beauchemin et al., 2018). We used the definition of descendants of immigrants from the French National Institute of Statistics and Economic Studies (INSEE, 2020) (i.e., included those who reported that at least one of their parents or two of their grand-parents from the same side were born outside Metropolitan France or Europe) to identify the category “racialized” in the French sample.

All sociodemographic characteristics were considered as potential confounders in our analysis because: a) they are empirically demonstrated explanatory factors (social determinants) associated with depression (Alegría et al., 2022; Jenkins et al., 2021; McQuaid et al., 2021; Silva et al., 2016), and b) are empirically or conceptually related to our exposure variable of income loss (de Miquel et al., 2022; Gama et al., 2021).

### 2.5 Statistical analysis

Characteristics of the study population were described for each survey, overall and compared by moderate-to-severe depressive symptoms (PHQ-9≥10), using Pearson’s Chi-squared test. A similar comparative analysis was performed to identify the main significant differences between the two study samples, using Pearson’s Chi-squared test. Multivariable logistic regression models were run to estimate the association between depressive symptoms and income loss, adjusted by all sociodemographic characteristics listed above, as well as country. To investigate the moderating effect of financial support on the association between income loss and depressive symptoms, two models were built for each survey cycle (2020, 2021) including an interaction term between income loss and financial support from government (i.e., Models 1 and 3), and an interaction term between income loss and financial support from family/friends (i.e., Models 2 and 4). As our main objective was to test the moderating effect of the financial support on depression among young adults who lost income due to COVID-19 – and not to investigate differences between Canada and France in terms of financial support interventions, all analyses presented were pooled by country, and a variable “country” was included in each model. The participant characteristics stratified by country are available in Supplementary Tables 1 and 2 Data analyses were performed using SAS software OnDemand for Academics.

## 3 Results

### 3.1 Selection of the study population

**Fig. 2:**
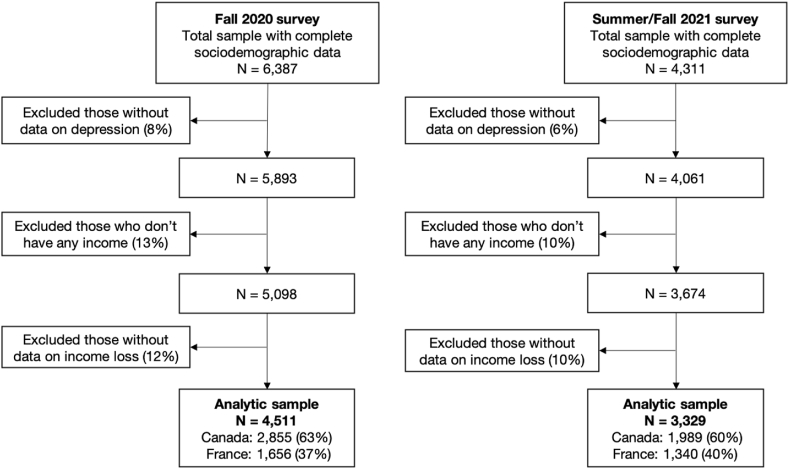
Flow chart of the study population (selected participants from the FOCUS Fall 2020 and Summer/Fall 2021 surveys).

Of the young adults enrolled in the Fall 2020 (n = 6,387) and Summer/Fall 2021 (n = 4,311) surveys with complete sociodemographic data, 8% (2020) and 6% (2021) were excluded from the present analysis because they did not complete the PHQ-9. Participants who did not report any individual income in the past year prior to the survey (i.e., 13% in Fall 2020 and 10% in Summer/Fall 2021) and those who had no data on income loss were also excluded (i.e., 12% in 2020 and 10% in 2021). The study population therefore included 4,511 (2020) and 3,329 (2021) young adults (see Fig. 2). In both surveys, about two-thirds of participants were from Canada (i.e., 63% in 2020, and 60% in 2021).

### 3.2 Participant characteristics

Characteristics of participants in both surveys are described in Table 1. Most sociodemographic characteristics were similar between surveys. Where not specified, results detailed hereafter in the text refer to the first Fall 2020 survey cycle. Overall, median age was 24 years (Interquartile Range [IQR] 21–26) in Fall 2020 and 25 years [IQR 22–27] in Summer/Fall 2021), with a higher proportion of participants aged 26–29 in the second survey (32.9% in 2020 versus 42.2% in 2021, p < 0.001). A total of 63.2% were women and 6.5% identified themselves as non-binary or with another gender identity than man or woman. Sixty-two percent of participants self-identified as straight/heterosexual, 18.6% were bisexual and 19.3% were from another sexual orientation. Seven percent reported being trans or were unsure about their trans identity. About half of participants were living in large cities (54.6%) and had some university educational attainment (49%), and one third were living with their family (29.4%). Some significant differences were observed between the two analysis samples. In the Fall 2020 survey, respectively 43.1% of participants were employed and 19.6% were students, while 53.8% were employed and 13.2% were students in the Summer/Fall 2021 survey (p < 0.001). A higher proportion of participants reported an annual income level ≥ CAD$ 20,000 in the second cycle compared to the first one (54.3% in 2021 versus 42.9% in 2020, p < 0.001). We also found a higher proportion of racialized young adults in the Summer/Fall 2021 survey (24.7% versus 13.3% in 2020, p < 0.001).

**Table 1:** Income loss, financial support, and sociodemographic characteristics of participants in the FOCUS Fall 2020 and Summer/Fall 2021 surveys; overall, and by moderate-to-severe depressive symptoms.

	Fall 2020					Summer/Fall 2021				
	Overall		Depressive symptoms (PHQ-9≥10)		p-value	Overall		Depressive symptoms (PHQ-9≥10)		p-value
	N	(col %)	N	(row %)		N	(col %)	N	(row %)	
**All participants**	4511	100	2413	53.5		3339	100	1520	45.6	
**Sociodemographic characteristics**										
**Country**					<0.001					<0.001
Canada	2855	63.3	1690	59.2		1989	59.6	1003	50.4	
France	1656	36.7	723	43.7		1340	40.1	517	38.6	
**Age (years)**					<0.001					<0.001
18–21	1287	28.5	812	63.1		706	21.1	364	51.6	
22–25	1739	38.6	918	52.8		1213	36.3	573	47.2	
26–29	1485	32.9	683	46		1410	42.2	583	41.3	
**Gender identity**					<0.001					<0.001
Man	1366	30.3	610	44.7		884	26.5	353	39.9	
Woman	2850	63.2	1579	55.4		2166	64.9	989	45.7	
Non-binary/other gender identity^a^	295	6.5	224	75.9		279	8.4	178	63.8	
**Trans identity**					<0.001					<0.001
Yes/unsure	302	6.7	221	73.2		267	8	187	70	
No	4209	93.3	2192	52.1		3062	91.7	1333	43.5	
**Sexual orientation**					<0.001					<0.001
Straight/heterosexual	2803	62.1	1322	47.2		2045	61.2	784	38.3	
Bisexual	839	18.6	547	65.2		584	17.5	334	57.2	
Other sexual identity^b^	869	19.3	544	62.6		700	21	402	57.4	
**Ethno-racial identity**					0.001					0.118
Non-racialized	3909	86.7	2054	52.5		2508	75.3	1125	44.9	
Racialized	602	13.3	359	59.6		821	24.7	394	48	
**Area of residence**					0.062					0.23
Large city	2464	54.6	1302	52.8		1740	52.1	807	46.4	
Medium city	965	21.4	548	56.8		743	22.3	348	46.8	
Small city or rural	1082	24	563	52		846	25.3	365	43.1	
**Educational attainment**					<0.001					<0.001
High school or college	1476	32.7	916	62.1		1038	31.1	568	54.7	
Some university	2212	49	1160	52.4		1583	47.4	700	44.2	
University graduate degree	823	18.2	337	40.9		708	21.2	252	35.6	
**Employment status**					<0.001					<0.001
Student	884	19.6	510	57.7		442	13.2	206	46.6	
Student and employed	1209	26.8	678	56.1		793	23.7	396	49.9	
Employed	1945	43.1	922	47.4		1797	53.8	738	41.1	
Unemployed	454	10.1	291	64.1		232	6.9	131	56.5	
Other situation	19	0.4	12	63.2		65	1.9	49	75.4	
**Individual income level (CAD)**					<0.001					<0.001
<$20,000	2368	52.5	1367	57.7		1385	41.5	709	51.2	
≥$20,000	1934	42.9	939	48.6		1813	54.3	748	41.3	
Missing data	209	4.6	107	51.2		131	3.9	63	48.1	
**Living arrangements**					<0.001					0.005
Alone	940	20.8	526	56		757	22.7	355	46.9	
With parents/family members	1328	29.4	758	57.1		834	25	410	49.2	
With partner	1304	28.9	614	47.1		1228	36.8	513	41.8	
With roommates, friends	939	20.8	515	54.8		510	15.3	242	47.5	
**Income loss**					<0.001					<0.001
All income loss	458	10.2	313	68.3		387	11.6	242	62.5	
Some income loss	1699	37.7	1036	61		721	21.6	378	52.4	
No income loss	2354	52.2	1064	45.2		2224	66.6	900	40.5	
**Governmental financial support**					<0.001					<0.001
Yes	1832	40.6	1084	59.2		592	17.7	328	55.4	
No	2679	59.4	1329	49.6		2737	82	1192	43.6	
**Family/friends financial support**					<0.001					<0.001
Yes	549	12.2	364	66.3		403	12.1	239	59.3	
No	3962	87.8	2049	51.7		2926	87.6	1281	43.8	
Governmental financial support*Income loss					<0.001					<0.001
*No governmental financial support*										
All income loss	175	3.9	116	66.3		245	5.4	150	61.2	
Some income loss	752	16.7	451	60		489	10.8	247	50.5	
No income loss	1752	38.8	762	43.5		2003	44.4	795	39.7	
*Government financial support*										
All income loss	283	6.3	197	69.6		139	3.1	92	66.2	
Some income loss	947	21	585	61.8		232	5.1	131	56.5	
No income loss	602	13.3	302	50.2		221	4.9	105	47.5	
Family/friends financial support*Income loss					<0.001					<0.001
*No family/friends financial support*										
All income loss	356	7.9	239	67.1		117	2.6	166	62.2	
Some income loss	1412	31.3	845	59.8		577	12.8	296	51.3	
No income loss	2194	48.6	965	44		2082	46.1	819	39.3	
*Family/friends financial support*										
All income loss	102	2.3	74	72.5		267	5.9	76	65	
Some income loss	287	6.4	191	66.5		144	3.2	82	56.9	
No income loss	160	3.5	99	61.9		142	3.1	81	57	

Notes. P-values were calculated from Pearson’s Chi-squared test.

a Other gender identity included intersex, Two-spirit (only for Canada), and other gender identity with an open-text box.

b Other sexual identity included asexual, pansexual, queer, Two-spirit (only for Canada) and other sexual identity with an open-text box.

### 3.3 Prevalence of depressive symptoms and financial supports

The prevalence of moderate-to-severe depressive symptoms was 53.5% in the Fall 2020 survey, and 45.6% in Summer/Fall 2021. In both cycles, participants from Canada experienced higher prevalence of moderate-to-severe depressive symptoms compared to French participants (59.2% versus 43.7% in 2020; 50.4% versus 38.6% in 2021). The prevalence of moderate-to-severe depressive symptoms was also higher in the following sociodemographic sub-groups: the youngest (18–21 years: 63.1% in 2020; 51.6% in 2021), gender and sexual minorities (e.g., non-binary/other gender identity: 75.9% in 2020; 63.8% in 2021), those with a low education level (high school or college: 62.1% in 2020; 54.7% in 2021), those unemployed (64.1% in 2020; 56.5% in 2021), and those with lower income level (<CAD$20,000: 57.7% in 2020; 51.2% in 2021). Those living with a partner had a lower prevalence of moderate-to-severe depressive symptoms compared to other groups (2020: 47.1% vs. >55%; 2021: 41.8% vs. >47%). While racialized participants had higher prevalence of depressive symptoms in the Fall 2020 cycle compared to non-racialized participants (59.6% versus 52.5%), this difference did not remain significant in the second cycle (48% versus 44.9%, p = 0.118). Similar prevalence of moderate-to-severe depressive symptoms was found in both cycles across areas of residence.

In the Fall 2020 cycle, 10.2% of participants reported having lost all, and 37.7% some, of their income due to the COVID-19 pandemic. More than two-thirds of these participants reported moderate-to-severe depressive symptoms (68.3% and 61% respectively). Lower prevalence rates of depressive symptoms were observed among those who did not report any income loss (45.2% in 2020 and 40.5% in 2021). In Fall 2020, 40.6% of participants received financial support from the government, with 59.2% of them having moderate-to-severe depressive symptoms, and 12.2% received financial support from family/friends, with 66.3% of this group having depressive symptoms. Only 5% received financial support from both the government and family/friends. In the Summer/Fall 2021 cycle, a lower proportion of participants indicated having lost some of their income in the last 6 months (21.6% versus 37.7% in 2020, p < 0.001) while a similar proportion reported having lost all their income (11.6%). Compared to Fall 2020, there were also fewer who received financial support from the government (40.6% versus 17.7%, p < 0.001) in Summer/Fall 2021, while a similar proportion received financial support from family/friends (12.1%). Only 3.5% received financial support from both the government and family/friends in Summer/Fall 2021. As in the first cycle, those having lost income (all: 62.5% and some income loss: 52.4% compared to those with no income loss: 40.5%) or reporting financial support from government or from family/friends experienced higher prevalence of moderate-to-severe depressive symptoms (government support: 55.4% vs. 43.6%; family support: 59.3% vs. 43.8%). When cross-tabulating participants according to income loss and financial support, those who lost all their income and did receive financial support, either from the government (69.6% in 2020; 66.2% in 2021) or from family/friends (72.5% in 2020; 65% in 2021) had the highest prevalence of depressive symptoms.

### 3.4 Moderators of financial support

**Fig. 3:**
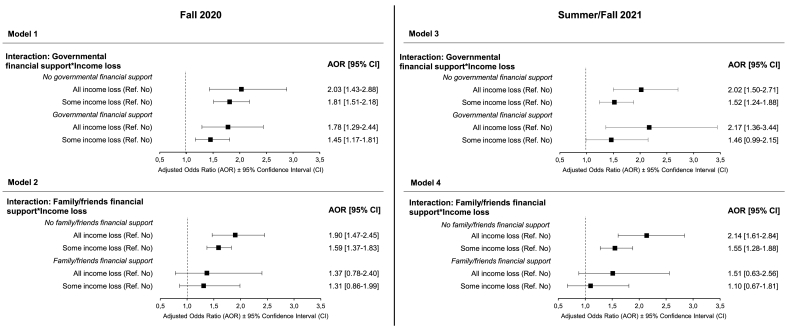
Multivariable regression models assessing the association between income loss and depressive symptoms, according to the type of financial support and adjusted on sociodemographic characteristics.

Among those who did not receive any financial support, income loss was associated with higher odds of reporting moderate-to-severe depressive symptoms, with adjustment for sociodemographic characteristics (Fig. 3; see Supplementary Table 3 for more details). Adjusted odds ratios (AOR) were greater for those who lost all their income as compared to those who only lost some income (e.g., Fall 2020, Model 1: AOR 2.03, 95% Confidence Interval [1.43–2.88]). In both cycles, among those who received governmental financial support, income loss remained significantly associated with moderate-to-severe depressive symptoms, whether participants lost all their income (Fall 2020, Model 1: AOR 1.75 [1.29–2.44]; Summer/Fall 2021, Model 3: AOR 2.17 [1.36–3.44]), or some of their income (Fall 2020, Model 1: AOR 1.45 [1.17–1.81]; Summer/Fall 2021, Model 3: AOR 1.46 [0.99–2.15]). However, among those who received financial support from family/friends, income loss was no longer significantly associated with moderate-to-severe depressive symptoms in both survey cycles, whether participants lost all their income (Fall 2020, Model 1: AOR 1.37 [0.78–2.40]; Summer/Fall 2021, Model 3: AOR 1.51 [0.88–2.56]), or some of their income (Fall 2020, Model 1: AOR 1.31 [0.86–1.99]; Summer/Fall 2021, Model 3: AOR 1.10 [0.67–1.81]).

## 4 Discussion

Our findings build upon an emerging body of research that documents the adverse effects of financial distress and economic uncertainty on mental health during the COVID-19 pandemic (Tham et al., 2021; Witteveen & Velthorst, 2020). Our study shows that financial support from family/friends moderates the negative effect of income loss due to the COVID-19 pandemic on depressive symptoms among young adults, such that those who lost income and received financial support reported lower risk of depressive symptoms than those who lost income and did not report any financial support. Effect measure modification by financial support from family/friends was more pronounced than that by government financial support, such that the AOR comparing moderate depressive symptoms by income loss was reduced but remained >1 and statistically significant among those who received the benefits from the government. These moderation effects were consistent across the two survey cycles conducted one year apart during the COVID-19 pandemic, highlighting the importance of continuing to provide financial support resources to young adults during and after public health crises.

Notwithstanding the positive and statistically significant AORs among those who received government benefits, our findings show that financial support may play a role in helping young adults cope with the ongoing pandemic-induced economic crisis and related mental health consequences. Data from a recent study in Canada offers potential explanations for this finding (Lindsay et al., 2021). For example, in this qualitative study conducted by Lindsay et al. (2021), youth and young adults ages 16–29 expressed that receiving financial support from the government (e.g., CERB) helped take the pressure off of finding employment. During the interviews, some participants also indicated that receiving financial support from their parents helped ease concerns related to the payment of their rent or groceries (Lindsay et al., 2021). Others receiving financial support from their parents also expressed less concern about finding a summer job to save money to return to university (Lindsay et al., 2021).

Our findings furthermore show that the magnitude of the moderation effect of financial support on the association between income loss and depression was dependent on the level of income loss related to the pandemic. With or without financial support, young adults who lost all their income still had higher risk for depression compared to those who reported having lost only some of their income. The detrimental consequences of facing COVID-19−induced economic hardship for mental health are most salient in the most economically vulnerable subgroups of young adults. Those who received financial support had a more difficult economic background and therefore may be more likely to report mental health issues. A previous study among adults in Canada showed that individuals who received government aid with a reduction in income were more likely to experience symptoms of psychological distress and anxiety, even though this was not significantly associated with symptoms of depression (Gill et al., 2022). Further efforts are needed to reinforce economic policies supporting young adults who are most financially impacted by the pandemic.

Finally, our findings provide insights on the importance of delivery mode of financial support with regard to its moderating effects. When adding the interaction terms with financial support from family/friends, the association between income loss and depressive symptoms were attenuated and became non-significant, suggesting a greater effect measure modification of family/friends financial support than the support from the government. Most of the financial support interventions made by the government of Canada and France were temporary, established to compensate the closures of business and the economic hardship during lockdown periods. In both countries, there were also numerous eligibility criteria that were required to access governmental funds (Government of Canada, 2021a; Government of France, 2021). Our study suggests that these governmental benefits may have been insufficient in magnitude and/or duration to have a pronounced effect on the mental well-being of young adults who lost income. Indeed, in Canada, the CERB has been criticized for its limitations, including its abolition before the end of economic-related public health restrictions and its lack of economic equity-related provisions (i.e., to maximize benefit for those in poverty) (Koebel et al., 2021). Conversely, financial support from family and friends may rely on a trusted and supportive relationship that provides timely and adapted economic relief. It may also feature a form of emotional support that could be considered as an additional critical coping strategy for young adults (Li et al., 2021; Mariani et al., 2020) – though we recognize not all youth have access to families/friends who are able to provide these kinds of support. Nevertheless, these findings suggest the need to provide diverse financial support initiatives to young adults with less barriers to structural and administrative aspects of obtaining the funds. Future studies should compare different funding support interventions to further disentangle the modalities and conditions that facilitate access young adults to financial support.

### 4.1 Strengths and limitations

Our study was conducted in two high-income countries among a large and diverse sample of young adults. This allowed us to include certain population characteristics (e.g., sexual and gender racialized youth, trans youth, unemployed youth) that have remained under-explored in other COVID-19 research. The repeated cross-sectional design enabled us to collect experiences of young adults at the two key periods of the pandemic (i.e., Fall 2020 and Summer/Fall 2021) where financial support interventions from the government were implemented. Compared to previous research on financial resources of young adults during the COVID-19 pandemic, our findings present two levels of income loss and two kinds of financial support, which provide a more in-depth understanding of the relationship between income loss, financial support, and their effects on depressive symptoms.

Although this study sheds light on the relationship between income loss, financial support and depression among young adults in the context of the COVID-19 pandemic, there are several limitations that may impact the interpretation of our findings. First, our study was comprised of a non-probabilistic sample and therefore may not be proportionally representative of the population of young adults living in Canada and France (e.g., our sample includes proportionately more women, as compared with national censuses). Despite this, our study sample included young adults from all provinces/regions in both countries, including provinces/regions that are more difficult to reach and often less represented in other research (e.g., Territories in Canada, Overseas in France). Moreover, as our primary objective was to examine the moderating effect of financial support on associations between income loss and depressive symptoms (rather than estimating prevalence); therefore, a representative sample is not necessary (Richiardi et al., 2013; Rothman et al., 2013). Furthermore, the statistical power of our analysis was not enough to detect a real difference in our modelling approach between the two type of financial supports. This methodological limitation underscores the need for future research examining the mental health benefits of socio-economic interventions, including the need to optimize various features of study designs in this area in order to more fulsomely assess the effect of financial support on mental health (e.g., sufficiently powered sample size; measures with better precision). Second, participant responses regarding their income loss and financial support may have been impacted by recall bias, as these measures were assessed over a period of six months prior to the time of survey, though arguably income loss is a significant and impactful event that one is unlikely to forget over the course of six months. Given that income loss and financial support were assessed over the same period of time (i.e., in the last 6 months), it was therefore not possible to determine whether study participants had received financial support before or after experiencing income loss. In addition, the exact levels of income loss and financial support were not collected, which limits our ability to fully understand at what level the financial support received from the government or from family/friends can compensate the loss of income related to the pandemic and its negative effects on mental health. Third, COVID-19-related financial supports that family/friends may have received (e.g., income supports, rent subsidies, allowances) were not collected as these supports primarily affected parent’s resources and not young adults directly. However, these changes may have influenced the ability of families and friends to provide financial support to young adults. To account for the participants’ overall economic background, all our regression models were adjusted on the individual income in the past year prior to the survey. Lastly, details about the emotional support from family and friends that young adults experienced, as well as pre-existing mental health conditions prior to the pandemic, were not collected and therefore not included in our analysis. This represents a limitation as both of these factors may have resulted in uncontrolled confounding of the association between income loss and depressive symptoms. It is indeed more likely that the beneficial effect of receiving emotional support from relatives may represent a key factor in the capacity of young adults to cope with stressful conditions associated with the onset of the COVID-19 pandemic, including the potential for this to mitigate the positive effect of receiving financial support on the risk of depressive symptoms. Nevertheless, this potential impact of uncontrolled confounding may have been mitigated by adjusting our analysis for a broad set of sociodemographic covariates.

### 4.2 Study implications

Our findings have implications for public health and social policies. Financial support interventions adopted during the pandemic might not only benefit the economic situation of young adults but can also help to mitigate the COVID-19-related mental health impact. However, effectively addressing the mental health issues of young adults during a pandemic remains a complex challenge that cannot be resolved by providing financial support alone. Rather, we suggest that financial supports contribute to a broader set of social determinants of mental health, which are often under-explored or resourced, and align with a comprehensive population-based mental health strategy. In addition to financial supports, other resources (e.g., campaign promoting positive mental health approach, trained providers, tailored mental health services) are needed that to best prevent and treat the full spectrum of mental health needs of young adult populations during and after periods of public health or economic crisis. In a survey on COVID-19 recovery policy preferences (Hammarberg et al., 2021), providing financial support for living expenses and mental health support were highly rated by more than 40% of young adults, indicating that continued financial support for young adults may be needed to avoid exacerbating existing inequalities regarding mental health. More research is needed to further assess the effect of financial support interventions that have been implemented by the governments to support young adults during the COVID-19 pandemic with a mental health perspective.

Further research is also needed to identify if there are other individual and structural moderators that can influence the association between income loss and mental health. A possible candidate could be social and emotional support; losing income might provoke a supporting reaction from a person’s environment, which, in turn, might lead to less mental health problems. For example, prior findings demonstrated that social support buffers the effect of unemployment on mental health (Milner et al., 2016), offering a positive environment to improve self-empowerment may help adopt effective coping strategies and reduce negative thoughts.

## 5 Conclusions

Our findings showed that the association between income loss and depression was moderated by receipt of family/friends financial support but not by receipt of government financial support. Our study also highlights that sub-groups of young adults who were financially most affected by the COVID-19 pandemic and related public health measures experienced higher rates of depressive symptoms. Adequate financial support interventions should be allocated to young adults during public health or economic crisis, especially among sub-groups who needed the most.

## Author contributions

**Pierre-julien Coulaud**: Conceptualization, Methodology, Validation, Investigation, Project administration, Data Curation, Writing - Original Draft, Writing - Review & Editing, Supervision. **Travis Salway**: Conceptualization, Methodology, Validation, Writing - Original Draft, Writing - Review & Editing. **Julie Jesson**: Conceptualization, Methodology, Formal analysis, Data Curation, Writing - Original Draft, Writing - Review & Editing. **Naseeb Bolduc**: Investigation, Project administration, Writing - Review & Editing. **Olivier Ferlatte**: Writing - Review & Editing. **Karine Bertrand**: Writing - Review & Editing. **Annabel Desgrées du Loû**: Writing - Review & Editing. **Emily Jenkins**: Writing - Review & Editing. **Marie Jauffret-Roustide**: Resources, Project administration, Writing - Review & Editing, Funding acquisition, Supervision. **Rod Knight**: Resources, Project administration, Writing - Review & Editing, Funding acquisition, Supervision.

## Funding

This study (The FOCUS Study) is supported by the 10.13039/501100000024Canadian Institutes of Health Research (CIHR, Funding Reference Numbers: VR5 172673 and AWD-017639) and by the 10.13039/501100001665French National Research Agency (ANR-21-COVR-011). The Cultural and Scientific Service of the Consulate General of French Embassy in Canada (Grant #F21-05226) also provided funds to support the open access publication of study results.

PJC is supported by a Postdoctoral Fellowship Award from the CIHR (Grant # MFE – 176609), as well as by funding received by RK from 10.13039/501100000024CIHR (VR5-172673 and AWD-017639). NB’s salary was supported by 10.13039/501100000024CIHR Grants VR5-172673 and AWD-017639. RK held a Scholar Awards from the 10.13039/501100000245Michael Smith Foundation for Health Research (Grant # 16808), which supported their time contributions to the study. The funders had no role in study design, data collection and analysis, decision to publish, or preparation of the manuscript.

## Ethical statement

Ethical approval was granted by the University of British Columbia Behavioural Research Ethics Board (H20-02053). Survey data was saved and stored in a secure server at the British Columbia Centre on Substance Use. In France, the Data Protection Officer at the University of Paris was consulted to ensure that our data privacy and security procedures were compliant with the European Union's General Data Protection Regulation (GDPR).

## Declaration of competing interest

The authors declare that they have no known competing financial interests or personal relationships that could have appeared to influence the work reported in this paper.

## Data Availability

Data will be made available on request.

## Abbreviations

AOR: Adjusted Odds Ratio
CAD: Canadian Dollars
CERB: Canada Emergency Response Benefit
CESB: Canada Emergency Student Benefit
CI: Confidence Intervals
EI: Employment Insurance
FOCUS: France Canada Observatory on COVID-19, Youth health and Social well-being
IQR: Interquartile Range
OECD: Organisation for Economic Co-operation and Development
PHQ-9: Patient Health Questionnaire-9
