# Supplementary material for: Moderation of the association between COVID-19-related income loss and depression by receipt of financial support: Repeated cross-sectional surveys of young adults in Canada and France (2020–2021)
Source: SSM Popul Health. 2023 Jan 11;21:101340. doi: 10.1016/j.ssmph.2023.101340 (PMC9832713; doi:10.1016/j.ssmph.2023.101340)
Supplement: Multimedia component 1 [file mmc1.docx]

**Supplementary Table 1. Study characteristics of participants living in Canada in the FOCUS Fall 2020 and Summer/Fall 2021 surveys; overall, and by moderate-to-severe depressive symptoms.**

|  |  | **Fall 2020** | | | | | |  | **Summer/Fall 2021** | | | | | |
| --- | --- | --- | --- | --- | --- | --- | --- | --- | --- | --- | --- | --- | --- | --- |
|  |  | **Overall** | |  | **Depressive**  **symptoms**  **(PHQ-9≥10)** | | **p-value** |  | **Overall** | |  | **Depressive symptoms (PHQ-9≥10)** | | **p-value** |
|  |  | **N** | **(col %)** |  | **N** | **(row %)** |  |  | **N** | **(col %)** |  | **N** | **(row %)** |  |
| **All participants** | | 2855 | 100 |  | 1690 | 59.2 |  |  | 1989 | 100 |  | 1003 | 50.4 |  |
| **Sociodemographic characteristics** | | | |  |  |  |  |  |  |  |  |  |  |  |
| **Age (years)** | |  |  |  |  |  | <0.001 |  |  |  |  |  |  | 0.024 |
|  | 18-21 | 849 | 29.7 |  | 583 | 68.7 |  |  | 440 | 22.1 |  | 242 | 55.0 |  |
|  | 22-25 | 1065 | 37.3 |  | 623 | 58.5 |  |  | 722 | 36.3 |  | 371 | 51.4 |  |
|  | 26-29 | 941 | 33 |  | 484 | 51.4 |  |  | 827 | 41.6 |  | 390 | 39 |  |
| **Gender identity** | |  |  |  |  |  | <0.001 |  |  |  |  |  |  | <0.001 |
|  | Man | 691 | 24.2 |  | 350 | 50.7 |  |  | 475 | 23.9 |  | 206 | 43.4 |  |
|  | Woman | 1931 | 67.6 |  | 1157 | 59.9 |  |  | 1296 | 65.2 |  | 647 | 65 |  |
|  | Non-binary/other gender identity^$^ | 233 | 8.2 |  | 183 | 78.5 |  |  | 218 | 11.0 |  | 150 | 68.8 |  |
| **Trans identity** | |  |  |  |  |  | <0.001 |  |  |  |  |  |  | <0.001 |
|  | Yes/unsure | 233 | 8.2 |  | 174 | 74.7 |  |  | 221 | 11.1 |  | 158 | 71.5 |  |
|  | No | 2622 | 91.8 |  | 1516 | 57.8 |  |  | 1768 | 88.9 |  | 845 | 47.8 |  |
| **Sexual orientation** | |  |  |  |  |  | <0.001 |  |  |  |  |  |  | <0.001 |
|  | Straight/heterosexual | 1635 | 57.3 |  | 851 | 52 |  |  | 1066 | 53.6 |  | 442 | 41.5 |  |
|  | Bisexual | 623 | 21.8 |  | 435 | 69.8 |  |  | 418 | 21.0 |  | 250 | 59.8 |  |
|  | Other sexual identity^£^ | 597 | 20.9 |  | 404 | 67.7 |  |  | 505 | 25.4 |  | 311 | 61.6 |  |
| **Ethno-racial identity** | |  |  |  |  |  | 0.006 |  |  |  |  |  |  | 0.058 |
|  | Non-racialized | 2456 | 86 |  | 1429 | 58.2 |  |  | 1644 | 82.7 |  | 813 | 49.5 |  |
|  | Racialized | 399 | 14 |  | 261 | 65.4 |  |  | 345 | 17.3 |  | 190 | 55.1 |  |
| **Area of residence** | |  |  |  |  |  | 0.004 |  |  |  |  |  |  | 0.74 |
|  | Large urban centre | 1590 | 55.7 |  | 907 | 57 |  |  | 1105 | 55.6 |  | 553 | 50.0 |  |
|  | Medium city or town | 621 | 21.8 |  | 402 | 64.7 |  |  | 434 | 21.8 |  | 226 | 52.1 |  |
|  | Rural area or small city | 644 | 22.6 |  | 381 | 59.2 |  |  | 450 | 22.6 |  | 224 | 49.8 |  |
| **Educational attainment** | |  |  |  |  |  | <0.001 |  |  |  |  |  |  | <0.001 |
|  | High school or college | 1025 | 35.9 |  | 687 | 67 |  |  | 754 | 37.9 |  | 434 | 57.6 |  |
|  | Some university | 1547 | 54.2 |  | 880 | 56.9 |  |  | 1046 | 52.6 |  | 491 | 46.9 |  |
|  | University graduate degree | 283 | 9.9 |  | 123 | 43.5 |  |  | 189 | 9.5 |  | 78 | 41.3 |  |
| **Employment status** | |  |  |  |  |  | <0.001 |  |  |  |  |  |  | <0.001 |
|  | Student | 500 | 17.5 |  | 315 | 63 |  |  | 220 | 11.1 |  | 116 | 52.7 |  |
|  | Student and employed | 821 | 28.8 |  | 505 | 61.5 |  |  | 574 | 28.9 |  | 284 | 49.5 |  |
|  | Employed | 1245 | 43.6 |  | 672 | 54.0 |  |  | 1053 | 52.9 |  | 502 | 47.7 |  |
|  | Unemployed | 274 | 9.6 |  | 188 | 68.6 |  |  | 98 | 4.9 |  | 64 | 65.3 |  |
|  | Other situation | 15 | 0.5 |  | 10 | 66.7 |  |  | 44 | 2.2 |  | 37 | 84.1 |  |
| **Individual income level (CAD)** | | |  |  |  |  | <0.001 |  |  |  |  |  |  | <0.001 |
|  | <$20,000 | 1377 | 48.2 |  | 884 | 64.2 |  |  | 716 | 36.0 |  | 409 | 57.1 |  |
|  | ≥$20,000 | 1345 | 47.1 |  | 736 | 54.7 |  |  | 1191 | 59.9 |  | 556 | 46.7 |  |
|  | Missing data | 133 | 4.7 |  | 70 | 52.6 |  |  | 82 | 4.1 |  | 38 | 46.3 |  |
| **Living arrangements** | |  |  |  |  |  | <0.001 |  |  |  |  |  |  | 0.45 |
|  | Alone | 400 | 14 |  | 268 | 67.0 |  |  | 330 | 16.6 |  | 173 | 52.4 |  |
|  | With parents/family members | 942 | 33 |  | 570 | 60.5 |  |  | 570 | 28.7 |  | 295 | 51.8 |  |
|  | With partner | 838 | 29.4 |  | 452 | 53.9 |  |  | 718 | 36.1 |  | 345 | 48.1 |  |
|  | With roommates, friends | 675 | 23.6 |  | 400 | 59.3 |  |  | 371 | 18.7 |  | 190 | 51.2 |  |
|  |  |  |  |  |  |  |  |  |  |  |  |  |  |  |
| **Income loss** | |  |  |  |  |  | <0.001 |  |  |  |  |  |  | <0.001 |
|  | All income loss | 328 | 11.5 |  | 237 | 72.3 |  |  | 236 | 11.9 |  | 161 | 68.2 |  |
|  | Some income loss | 1215 | 42.6 |  | 774 | 63.7 |  |  | 490 | 24.6 |  | 271 | 55.3 |  |
|  | No income loss | 1312 | 46 |  | 679 | 51.8 |  |  | 1263 | 63.5 |  | 571 | 45.2 |  |
| **Governmental financial support** | | | |  |  |  | <0.001 |  |  |  |  |  |  | <0.001 |
|  | Yes | 1520 | 53.2 |  | 952 | 62.6 |  |  | 492 | 24.7 |  | 286 | 58.1 |  |
|  | No | 1335 | 46.8 |  | 738 | 55.3 |  |  | 1497 | 75.3 |  | 717 | 47.9 |  |
| **Family/friends financial support** | | | |  |  |  | <0.001 |  |  |  |  |  |  | <0.001 |
|  | Yes | 325 | 11.4 |  | 233 | 71.7 |  |  | 238 | 12 |  | 158 | 66.4 |  |
|  | No | 2530 | 88.6 |  | 1457 | 57.6 |  |  | 1751 | 88 |  | 845 | 48.3 |  |
| **Governmental financial support*Income loss** | | | | | |  | <0.001 |  |  |  |  |  |  | <0.001 |
| *No governmental financial support* | | | |  |  |  |  |  |  |  |  |  |  |  |
|  | All income loss | 69 | 2.4 |  | 49 | 71 |  |  | 119 | 6 |  | 82 | 68.9 |  |
|  | Some income loss | 364 | 12.8 |  | 237 | 32 |  |  | 287 | 14.4 |  | 152 | 53 |  |
|  | No income loss | 902 | 31.6 |  | 452 | 50.1 |  |  | 1091 | 54.8 |  | 483 | 44.3 |  |
| *Government financial support* | | |  |  |  |  |  |  |  |  |  |  |  |  |
|  | All income loss | 259 | 9.1 |  | 188 | 72.6 |  |  | 117 | 5.9 |  | 79 | 67.5 |  |
|  | Some income loss | 851 | 29.8 |  | 537 | 63.1 |  |  | 203 | 10.2 |  | 119 | 51.2 |  |
|  | No income loss | 410 | 14.4 |  | 227 | 55.4 |  |  | 172 | 8.7 |  | 88 | 58.6 |  |
| **Family/friends financial support*Income loss** | | | | | |  | <0.001 |  |  |  |  |  |  | <0.001 |
| *No family/friends financial support* | | | |  |  |  |  |  |  |  |  |  |  |  |
|  | All income loss | 262 | 9.2 |  | 187 | 71.4 |  |  | 170 | 8.6 |  | 110 | 64.7 |  |
|  | Some income loss | 1040 | 36.4 |  | 651 | 62.6 |  |  | 400 | 20.1 |  | 216 | 54 |  |
|  | No income loss | 1228 | 43.0 |  | 619 | 50.4 |  |  | 1181 | 59.4 |  | 519 | 44 |  |
| *Family/friends financial support* | | | |  |  |  |  |  |  |  |  |  |  |  |
|  | All income loss | 66 | 2.3 |  | 50 | 75.8 |  |  | 66 | 3.3 |  | 51 | 77.3 |  |
|  | Some income loss | 175 | 6.1 |  | 123 | 70.3 |  |  | 90 | 4.5 |  | 55 | 61.1 |  |
|  | No income loss | 84 | 2.9 |  | 60 | 71.4 |  |  | 82 | 4.1 |  | 52 | 63.4 |  |
| *Notes. P-values were calculated from Pearson’s Chi-squared test.* | | | | | | |  |  |  |  |  |  |  |  |
| *^$^Other gender identity included intersex, Two-spirit, and other gender identity with an open-text box.* | | | | | | | | | | | | |  |  |
| *^£^Other sexual identity included asexual, pansexual, queer, Two-spirit and other sexual identity with an open-text box.* | | | | | | | | | | | | | | |

**Supplementary Table 2. Study characteristics of participants living in France in the FOCUS Fall 2020 and Summer/Fall 2021 surveys; overall, and by moderate-to-severe depressive symptoms.**

|  |  | **Fall 2020** | | | | | |  | **Summer/Fall 2021** | | | | | |
| --- | --- | --- | --- | --- | --- | --- | --- | --- | --- | --- | --- | --- | --- | --- |
|  |  | **Overall** | |  | **Depressive**  **symptoms**  **(PHQ-9≥10)** | | **p-value** |  | **Overall** | |  | **Depressive symptoms (PHQ-9≥10)** | | **p-value** |
|  |  | **N** | **(col %)** |  | **N** | **(row %)** |  |  | **N** | **(col %)** |  | **N** | **(row %)** |  |
| **All participants** | | 1656 | 100 |  | 723 | 43.7 |  |  | 1340 | 100 |  | 516 | 38.5 |  |
| **Sociodemographic characteristics** | | | |  |  |  |  |  |  |  |  |  |  |  |
| **Age (years)** | |  |  |  |  |  | <0.001 |  |  |  |  |  |  | 0.001 |
|  | 18-21 | 438 | 26.4 |  | 229 | 52.3 |  |  | 266 | 19.9 |  | 122 | 45.9 |  |
|  | 22-25 | 674 | 40.7 |  | 295 | 43.8 |  |  | 491 | 36.6 |  | 201 | 40.9 |  |
|  | 26-29 | 544 | 32.9 |  | 199 | 36.6 |  |  | 583 | 43.5 |  | 193 | 33.1 |  |
| **Gender identity** | |  |  |  |  |  | <0.001 |  |  |  |  |  |  | 0.24 |
|  | Man | 675 | 40.8 |  | 260 | 38.5 |  |  | 410 | 30.6 |  | 147 | 35.9 |  |
|  | Woman | 919 | 55.5 |  | 422 | 45.9 |  |  | 869 | 64.9 |  | 341 | 39.2 |  |
|  | Non-binary/other gender identity^$^ | 62 | 3.7 |  | 41 | 66.1 |  |  | 61 | 4.6 |  | 28 | 45.9 |  |
| **Trans identity** | |  |  |  |  |  | <0.001 |  |  |  |  |  |  | <0.001 |
|  | Yes/unsure | 69 | 4.2 |  | 47 | 68.1 |  |  | 46 | 3.4 |  | 29 | 63 |  |
|  | No | 1587 | 95.8 |  | 676 | 42.6 |  |  | 1294 | 96.6 |  | 487 | 37.6 |  |
| **Sexual orientation** | |  |  |  |  |  | <0.001 |  |  |  |  |  |  | <0.001 |
|  | Straight/heterosexual | 1168 | 70.5 |  | 471 | 40.3 |  |  | 979 | 73.1 |  | 341 | 34.8 |  |
|  | Bisexual | 216 | 13.0 |  | 112 | 51.9 |  |  | 166 | 12.4 |  | 84 | 50.6 |  |
|  | Other sexual identity^£^ | 272 | 16.4 |  | 140 | 51.5 |  |  | 195 | 14.6 |  | 91 | 46.7 |  |
| **Ethno-racial identity** | |  |  |  |  |  | 0.16 |  |  |  |  |  |  | 0.015 |
|  | Non-racialized | 1453 | 87.7 |  | 625 | 43 |  |  | 864 | 64.5 |  | 312 | 36.1 |  |
|  | Racialized | 203 | 12.3 |  | 98 | 48.3 |  |  | 476 | 35.5 |  | 204 | 42.9 |  |
| **Area of residence** | |  |  |  |  |  | 0.40 |  |  |  |  |  |  | 0.33 |
|  | Large urban centre | 874 | 52.8 |  | 395 | 45.2 |  |  | 636 | 47.5 |  | 254 | 39.9 |  |
|  | Medium city or town | 344 | 20.8 |  | 146 | 42.4 |  |  | 309 | 23.1 |  | 122 | 39.5 |  |
|  | Rural area or small city | 438 | 26.4 |  | 182 | 41.6 |  |  | 395 | 29.5 |  | 140 | 35.4 |  |
| **Educational attainment** | |  |  |  |  |  | 0.001 |  |  |  |  |  |  | <0.001 |
|  | High school or college | 451 | 27.2 |  | 229 | 50.8 |  |  | 285 | 21.3 |  | 134 | 47 |  |
|  | Some university | 665 | 40.2 |  | 280 | 42.1 |  |  | 536 | 40 |  | 208 | 38.8 |  |
|  | University graduate degree | 540 | 32.6 |  | 214 | 39.6 |  |  | 519 | 38.7 |  | 174 | 33.5 |  |
| **Employment status** | |  |  |  |  |  | <0.001 |  |  |  |  |  |  | <0.001 |
|  | Student | 384 | 23.2 |  | 195 | 50.8 |  |  | 221 | 16.5 |  | 89 | 40.3 |  |
|  | Student and employed | 388 | 23.4 |  | 173 | 44.6 |  |  | 219 | 16.3 |  | 112 | 51.1 |  |
|  | Employed | 700 | 42.3 |  | 250 | 35.7 |  |  | 745 | 55.6 |  | 236 | 31.7 |  |
|  | Unemployed | 180 | 10.9 |  | 103 | 57.2 |  |  | 134 | 10 |  | 67 | 50 |  |
|  | Other situation | 4 | 0.2 |  | 2 | 50 |  |  | 21 | 1.6 |  | 12 | 57.1 |  |
| **Individual income level (CAD)** | | |  |  |  |  | <0.001 |  |  |  |  |  |  | <0.001 |
|  | <$20,000 | 991 | 59.8 |  | 483 | 48.7 |  |  | 669 | 49.9 |  | 299 | 44.7 |  |
|  | ≥$20,000 | 589 | 35.6 |  | 203 | 34.5 |  |  | 622 | 46.4 |  | 192 | 30.9 |  |
|  | Missing data | 76 | 4.6 |  | 37 | 48.7 |  |  | 49 | 3.7 |  | 25 | 51 |  |
| **Living arrangements** | |  |  |  |  |  | <0.001 |  |  |  |  |  |  | 0.006 |
|  | Alone | 540 | 32.6 |  | 258 | 47.8 |  |  | 427 | 31.9 |  | 182 | 42.6 |  |
|  | With parents/family members | 386 | 23.3 |  | 188 | 48.7 |  |  | 263 | 19.6 |  | 114 | 43.3 |  |
|  | With partner | 466 | 28.1 |  | 162 | 34.8 |  |  | 511 | 38.1 |  | 168 | 32.9 |  |
|  | With roommates, friends | 264 | 15.9 |  | 115 | 43.6 |  |  | 139 | 10.4 |  | 52 | 37.4 |  |
|  |  |  |  |  |  |  |  |  |  |  |  |  |  |  |
| **Income loss** | |  |  |  |  |  | <0.001 |  |  |  |  |  |  | <0.001 |
|  | All income loss | 130 | 7.9 |  | 76 | 58.5 |  |  | 148 | 11 |  | 81 | 54.7 |  |
|  | Some income loss | 484 | 29.2 |  | 262 | 54.1 |  |  | 231 | 17.2 |  | 107 | 46.3 |  |
|  | No income loss | 1042 | 62.9 |  | 385 | 36.9 |  |  | 961 | 71.7 |  | 328 | 34.1 |  |
| **Governmental financial support** | | | |  |  |  | 0.59 |  |  |  |  |  |  | 0.46 |
|  | Yes | 312 | 18.8 |  | 132 | 42.3 |  |  | 100 | 7.5 |  | 42 | 42 |  |
|  | No | 1344 | 81.2 |  | 591 | 44 |  |  | 1240 | 92.5 |  | 474 | 38.2 |  |
| **Family/friends financial support** | | | |  |  |  | <0.001 |  |  |  |  |  |  | 0.003 |
|  | Yes | 224 | 13.5 |  | 131 | 58.5 |  |  | 165 | 12.3 |  | 81 | 49.1 |  |
|  | No | 1432 | 86.5 |  | 592 | 41.3 |  |  | 1175 | 87.7 |  | 435 | 37 |  |
| **Governmental financial support*Income loss** | | | | | |  | <0.001 |  |  |  |  |  |  | <0.001 |
| *No governmental financial support* | | | |  |  |  |  |  |  |  |  |  |  |  |
|  | All income loss | 106 | 6.4 |  | 67 | 63.2 |  |  | 126 | 9.4 |  | 68 | 54 |  |
|  | Some income loss | 388 | 23.4 |  | 214 | 55.1 |  |  | 202 | 15.1 |  | 95 | 47 |  |
|  | No income loss | 850 | 51.3 |  | 310 | 36.5 |  |  | 912 | 68.1 |  | 311 | 34.1 |  |
| *Government financial support* | | |  |  |  |  |  |  |  |  |  |  |  |  |
|  | All income loss | 24 | 1.4 |  | 9 | 37.5 |  |  | 22 | 1.6 |  | 13 | 59.1 |  |
|  | Some income loss | 96 | 5.8 |  | 48 | 50 |  |  | 29 | 2.2 |  | 12 | 41.4 |  |
|  | No income loss | 192 | 11.6 |  | 75 | 39.1 |  |  | 49 | 3.7 |  | 17 | 34.7 |  |
| **Family/friends financial support*Income loss** | | | | | |  | <0.001 |  |  |  |  |  |  | <0.001 |
| *No family/friends financial support* | | | |  |  |  |  |  |  |  |  |  |  |  |
|  | All income loss | 94 | 5.7 |  | 52 | 55.3 |  |  | 97 | 7.2 |  | 56 | 57.7 |  |
|  | Some income loss | 372 | 22.5 |  | 194 | 52.2 |  |  | 177 | 13.2 |  | 80 | 45.2 |  |
|  | No income loss | 966 | 58.3 |  | 346 | 35.8 |  |  | 901 | 67.2 |  | 299 | 33.2 |  |
| *Family/friends financial support* | | | |  |  |  |  |  |  |  |  |  |  |  |
|  | All income loss | 36 | 2.2 |  | 24 | 66.7 |  |  | 51 | 3.8 |  | 25 | 49.0 |  |
|  | Some income loss | 112 | 6.8 |  | 68 | 60.7 |  |  | 54 | 4 |  | 27 | 50 |  |
|  | No income loss | 76 | 4.6 |  | 39 | 51.3 |  |  | 60 | 4.5 |  | 29 | 48.3 |  |
| *Notes. P-values were calculated from Pearson’s Chi-squared test.* | | | | | | |  |  |  |  |  |  |  |  |
| *^$^Other gender identity included intersex, and other gender identity with an open-text box.* | | | | | | | | | | | | |  |  |
| *^£^Other sexual identity included asexual, pansexual, queer, and other sexual identity with an open-text box.* | | | | | | | | | | | | | | |

**Supplementary Table 3. Detailed findings of the multivariable regression models assessing the association between income loss and depressive symptoms, according to the type of financial support and adjusted on sociodemographic characteristics.**

|  |  | **Fall 2020** | | | | |  | **Summer/Fall 2021** | | | | |
| --- | --- | --- | --- | --- | --- | --- | --- | --- | --- | --- | --- | --- |
|  |  | **Depressive symptoms (Model 1)** | |  | **Depressive symptoms (Model 2)** | |  | **Depressive symptoms (Model 3)** | |  | **Depressive symptoms (Model 4)** | |
|  |  | **AOR** | **(95% CIs)^1^** |  | **AOR** | **(95% CIs)^1^** |  | **AOR** | **(95% CIs)^1^** |  | **AOR** | **(95% CIs)^1^** |
| **Interaction: Income loss * Governmental financial support** | | |  |  |  |  |  |  |  |  |  |  |
| *No governmental financial support* | |  |  |  |  |  |  |  |  |  |  |  |
|  | All income loss (Ref. No income loss) | 2.03 | (1.43-2.88) |  |  |  |  | 2.02 | (1.50-2.71) |  |  |  |
|  | Some income loss (Ref. No income loss) | 1.81 | (1.51-2.18) |  |  |  |  | 1.52 | (1.24-1.88) |  |  |  |
| *Governmental financial support* | |  |  |  |  |  |  |  |  |  |  |  |
|  | All income loss (Ref. No income loss) | 1.78 | (1.29-2.44) |  |  |  |  | 2.17 | (1.36-3.44) |  |  |  |
|  | Some income loss (Ref. No income loss) | 1.45 | (1.17-1.81) |  |  |  |  | 1.46 | (0.99-2.15) |  |  |  |
| **Interaction: Income loss * Family/friends financial support** | | |  |  |  |  |  |  |  |  |  |  |
| *No family/friends financial support* | |  |  |  |  |  |  |  |  |  |  |  |
|  | All income loss (Ref. No income loss) |  |  |  | 1.9 | (1.47-2.45) |  |  |  |  | 2.14 | (1.61-2.84) |
|  | Some income loss (Ref. No income loss) |  |  |  | 1.59 | (1.37-1.83) |  |  |  |  | 1.55 | (1.28-1.88) |
| *Family/friends financial support* | |  |  |  |  |  |  |  |  |  |  |  |
|  | All income loss (Ref. No income loss) |  |  |  | 1.37 | (0.78-2.40) |  |  |  |  | 1.51 | (0.88-2.56) |
|  | Some income loss (Ref. No income loss) |  |  |  | 1.31 | (0.86-1.99) |  |  |  |  | 1.1 | (0.67-1.81) |
| **Sociodemographic characteristics** | |  |  |  |  |  |  |  |  |  |  |  |
| **Country (Ref. Canada)** | |  |  |  |  |  |  |  |  |  |  |  |
|  | France | 0.59 | (0.50-0.68) |  | 0.6 | (0.52-0.70) |  | 0.74 | (0.62-0.89) |  | 0.73 | (0.61-0.87) |
| **Age (Ref. 18-21 years)** | |  |  |  |  |  |  |  |  |  |  |  |
|  | 22-25 | 0.78 | (0.66-0.93) |  | 0.77 | (0.64-0.91) |  | 0.99 | (0.79-1.22) |  | 0.99 | (0.79-1.23) |
|  | 26-29 | 0.68 | (0.55-0.84) |  | 0.67 | (0.54-0.83) |  | 0.92 | (0.72-1.18) |  | 0.92 | (0.72-1.18) |
| **Gender identity (Ref. Man)** | |  |  |  |  |  |  |  |  |  |  |  |
|  | Woman | 1.52 | (1.31-1.75) |  | 1.5 | (1.30-1.73) |  | 1.35 | (1.13-1.60) |  | 1.35 | (1.13-1.61) |
|  | Non-binary/other gender identity*^$^* | 2.09 | (1.43-3.05) |  | 2.11 | (1.44-3.08) |  | 1.08 | (0.74-1.56) |  | 1.10 | (0.76-1.59) |
| **Trans identity (Ref. No)** | |  |  |  |  |  |  |  |  |  |  |  |
|  | Yes/unsure | 1.32 | (0.93-1.89) |  | 1.32 | (0.92-1.88) |  | 2.16 | (1.48-3.14) |  | 2.12 | (1.45-3.08) |
| **Sexual orientation (Ref. Straight/heterosexual)** | |  |  |  |  |  |  |  |  |  |  |  |
|  | Bisexual | 1.62 | (1.37-1.93) |  | 1.61 | (1.36-1.91) |  | 1.70 | (1.39-2.08) |  | 1.68 | (1.38-2.06) |
|  | Other sexual minority*^£^* | 1.41 | (1.18-1.69) |  | 1.39 | (1.16-1.66) |  | 1.59 | (1.30-1.95) |  | 1.59 | (1.30-1.95) |
| **Ethno-racial identity (Ref. Non-racialized)** | |  |  |  |  |  |  |  |  |  |  |  |
|  | Racialized | 1.25 | (1.04-1.51) |  | 1.25 | (1.04-1.51) |  | 1.17 | (0.98-1.39) |  | 1.16 | (0.98-1.38) |
| **Area of residence (Ref. Large urban centre)** | |  |  |  |  |  |  |  |  |  |  |  |
|  | Medium city or town | 1.08 | (0.92-1.27) |  | 1.08 | (0.92-1.27) |  | 1.04 | (0.86-1.25) |  | 1.03 | (0.86-1.24) |
|  | Rural area or small city | 1.04 | (0.88-1.21) |  | 1.06 | (0.90-1.24) |  | 0.9 | (0.75-1.08) |  | 0.90 | (0.75-1.09) |
| **Educational attainment (Ref. Some University)** | |  |  |  |  |  |  |  |  |  |  |  |
|  | High school or college | 1.35 | (1.16-1.57) |  | 1.35 | (1.16-1.57) |  | 1.48 | (1.24-1.77) |  | 1.47 | (1.23-1.75) |
|  | University graduate degree | 0.93 | (0.77-1.12) |  | 0.94 | (0.78-1.13) |  | 0.97 | (0.79-1.20) |  | 0.97 | (0.79-1.20) |
| **Employment status (Ref. Employed)** | |  |  |  |  |  |  |  |  |  |  |  |
|  | Student | 1.18 | (0.96-1.44) |  | 1.14 | (0.94-1.40) |  | 1.01 | (0.79-1.3) |  | 1.01 | (0.78-1.29) |
|  | Student and employed | 1.09 | (0.91-1.31) |  | 1.07 | (0.90-1.28) |  | 1.1 | (0.90-1.35) |  | 1.11 | (0.90-1.36) |
|  | Unemployed | 1.67 | (1.32-2.12) |  | 1.61 | (1.27-2.04) |  | 1.39 | (1.02-1.88) |  | 1.38 | (1.01-1.86) |
| **Individual income (Ref.** **≥$20,000)** | |  |  |  |  |  |  |  |  |  |  |  |
|  | <$20,000 | 1.09 | (0.93-1.28) |  | 1.06 | (0.91-1.25) |  | 1.16 | (0.97-1.40) |  | 1.15 | (0.95-1.38) |
|  | Missing data | 0.82 | (0.60-1.12) |  | 0.81 | (0.59-1.11) |  | 1.15 | (0.79-1.69) |  | 1.13 | (0.77-1.66) |
| **Living arrangements (Ref. living with parents/family members)** | | |  |  |  |  |  |  |  |  |  |  |
|  | Alone | 1.55 | (1.28-1.89) |  | 1.53 | (1.26-1.86) |  | 1.32 | (1.06-1.66) |  | 1.3 | (1.04-1.63) |
|  | Living with partner | 0.9 | (0.75-1.07) |  | 0.88 | (0.74-1.06) |  | 0.96 | (0.78-1.18) |  | 0.96 | (0.78-1.18) |
|  | Living with roommates or friends | 0.97 | (0.81-1.17) |  | 0.96 | (0.80-1.15) |  | 1 | (0.79-1.28) |  | 1 | (0.78-1.27) |

*Notes. Statistically significant associations (p < 0.05) are highlighted in bold.*

*^1^AOR = Adjusted Odds Ratio, CI = Confidence Interval.*

*^$^Other gender identity included intersex, Two-spirit (only for Canada), and other gender identity with an open-text box.*

*^£^Other sexual identity included asexual, pansexual, queer, Two-spirit (only for Canada) and other sexual identity with an open-text box.*
